# The Mobility of Eurasian Avian-like M2 Is Determined by Residue E79 Which Is Essential for Pathogenicity of 2009 Pandemic H1N1 Influenza Virus in Mice

**DOI:** 10.3390/v15122365

**Published:** 2023-11-30

**Authors:** Rujuan Wu, Xinyu Zeng, Mingqing Wu, Lixiang Xie, Guanlong Xu, Yaqing Mao, Zhaofei Wang, Yuqiang Cheng, Heng’an Wang, Yaxian Yan, Jianhe Sun, Jingjiao Ma

**Affiliations:** 1 Shanghai Key Laboratory of Veterinary Biotechnology, Key Laboratory of Urban Agriculture (South), Ministry of Agriculture, School of Agriculture and Biology, Shanghai Jiao Tong University, Shanghai 200240, China; wrujuan9425@163.com (R.W.); zengxydb@sjtu.edu.cn (X.Z.); shixiang@sjtu.edu.cn (M.W.); xielixiang2020@gmail.com (L.X.); wzfxlzjx@sjtu.edu.cn (Z.W.); wyycyq@sjtu.edu.cn (Y.C.); hawang@sjtu.edu.cn (H.W.); yanyaxian@sjtu.edu.cn (Y.Y.); 2 Ganzhou Polytechnic, Ganzhou 341000, China; 3 China Institute of Veterinary Drug Control, Beijing 100081, China; xuguanlongw@163.com (G.X.); wcpmyq@163.com (Y.M.)

**Keywords:** influenza M2, (H1N1)pdm09, pathogenicity, proinflammatory response, NLRP3

## Abstract

In 2009, a novel H1N1 influenza virus caused the first influenza pandemic of the 21st century. Studies have shown that the influenza M gene played important roles in the pathogenicity and transmissibility of the 2009 H1N1 pandemic ((H1N1)pdm09), whilst the underlying mechanism remains unclear. The influenza M gene encodes two proteins, matrix protein 1 and matrix protein 2, which play important roles in viral replication and assembly. In this study, it is found that the M2 protein of the (H1N1)pdm09 virus showed a lower mobility rate than the North America triple-reassortant influenza M2 protein in Polyacrylamide Gel Electrophoresis (PAGE). The site-directed mutations of the amino acids of (H1N1)pdm09 M2 revealed that E79 is responsible for the mobility rate change. Further animal studies showed that the (H1N1)pdm09 containing a single M2-E79K was significantly attenuated compared with the wild-type virus in mice and induced lower proinflammatory cytokines and IFNs in mouse lungs. Further in vitro studies indicated that this mutation also affected NLRP3 inflammasome activation. To reveal the reason why they have different mobility rates, a circular dichroism spectra assay was employed and showed that the two M2 proteins displayed different secondary structures. Overall, our findings suggest that M2 E79 is important for the virus replication and pathogenicity of (H1N1)pdm09 through NLRP3 inflammasome and proinflammatory response.

## 1. Introduction

Influenza A virus (IAV), a member of the *Orthomyxoviridae* family, poses a significant threat to public health worldwide [1,2,3]. To date, 18 HA subtypes and 11 NA subtypes of IAVs have been identified [4,5]. Among these subtypes, only H1, H2, and H3 have caused pandemics in humans in the past, and certain avian influenza viruses (for example H5N1 and H7N9) have only caused endemic outbreaks without readily transmitting from human to human [6]. In early April 2009, a new pandemic influenza virus ((H1N1)pdm09) was reported in Mexico and the United States [7]. This virus subsequently spread globally within months and caused the first influenza pandemic of the 21st century, as declared by the World Health Organization (WHO) on 11 June 2009. The virus quickly adapted to humans and exhibited global transmission, resulting in over 503,536 human cases worldwide, including approximately 20,000 deaths. It was characterized by its rapid spread and tendency to infect young people [8].

Genetic analysis indicates that the (H1N1)pdm09 virus is a triple-reassortant virus that contains gene segments from viruses of different origins. The PB2/PA (originally from avian influenza virus), PB1 (originally from human H3N2), and HA/NP/NS (originally from classical swine H1N1 virus) genes were derived from a North American triple-reassortant swine influenza virus [9]. Interestingly, the NA and M gene segments are from a Eurasian avian-like swine H1N1 lineage that had not been reported in North America before 2009. Further studies indicated that the combination of Eurasian-origin NA and M genes contributed to the transmissibility of the (H1N1)pdm09 virus by enhancing the release of influenza viral RNA-containing particles into the air [10,11]. However, the exact role of the NA and M genes in the pathogenicity of the (H1N1)pdm09 virus remains largely unclear. (H1N1)pdm09 transmitted into many different species and is recombinant with domestic viruses, especially in pigs, which are called “mixing vessels,” resulting in variant viruses. Based on many genetic studies, almost all the variant viruses contain the M gene from (H1N1)pdm09 [12]. The variant swine influenza viruses are able to infect humans and cause death [13]. In our previous study, genetically different swine influenza viruses passed in pigs also showed that variant viruses containing the M gene are viable in pigs. Studies have confirmed that the M gene of (H1N1)pdm09 is important for virus morphology, aerosol transmission, and pathology. John Steel et al. reported that the introduction of a single M gene of (H1N1)pdm09 improved viral transmissibility in the PR8/H1N1 background, emphasizing the important role of the M gene of the (H1N1)pdm09 virus [14]. Further study conducted by the same group indicated that residue 41 of the Eurasian avian-like M1 gene contributes to the improved transmissibility of the (H1N1)pdm09 virus [15]. Other studies have shown that the (H1N1)pdm09 M gene increases swine influenza viral transmissibility among mammals. However, the underlying mechanism remains unclear [16].

The influenza virus M gene encodes two proteins, namely matrix protein 1 (M1) and matrix protein 2 (M2). M1 has multiple functions in viral assembly, budding, and morphology; forms the viral capsid; and interacts with other viral proteins such as neuraminidase (NA) and hemagglutinin (HA), which are crucial for viral pathogenesis [17,18]. M2 is a viral ion-channel protein [19], embedded in the viral envelope that serves multiple functions. During viral budding, M1, HA, and NA proteins are localized on the lipid raft of the cell membrane, and the M2 protein bends the membrane to form the virion [20]. The ion-channel has been reported activates the NOD-like receptor family pyrin domain-containing 3 (NLRP3) inflammasome during influenza virus infection, which causes inflammation and diseases; treatment with an NLRP3 inhibitor after infection improved disease outcome without impairing viral clearance [21,22]. Influenza virus infection stimulates the inflammasome through NLRP3, which forms a complex with ASC and caspase-1. The activation of TLRs or RLRs leads to the upregulation of pro-forms of interleukin-1β (IL-1β) and IL-18. The activation of the inflammasome recruits pro-caspase-1 and subsequently produces mature IL-1β and IL-18 [22,23], whilst the role of the M2 protein in the pathogenicity of (H1N1)pdm09 virus remains unclear.

In our study, we observed that the M2 proteins of Eurasia avian-like and North America triple-reassortant swine influenza viruses showed varied molecular weights in Polyacrylamide Gel Electrophoresis, although the two proteins contain the same amino acid numbers. Further study showed that the mobility rate change of M2 is determined by the amino acid residue at position 79. The Eurasia M2 containing E79 showed a larger molecular weight than that of the North America triple-reassortant swine influenza virus containing K79. Introduction of the E79K mutation reduced the molecular weight of the (H1N1)pdm09 M2 protein. Interestingly, an animal study showed that the CA09-M2-E79K virus with the M2 E79K mutation results in decreased replication and virulence in mouse models. Analysis of cytokine levels in mouse lungs revealed that the CA09-M2-E79K infection induces less interferons (IFN) and inflammation response in the early stage (3 dpi) of infection, which suggests that the M2 E79 residue is important for proinflammatory response of CA09 in mouse models. Our study demonstrated that residue E79 of the M2 protein plays an essential role in the replication and pathogenicity of the (H1N1)pdm09 virus.

## 2. Materials and Methods

### 2.1. Cells

Madin-Darby canine kidney (MDCK) cells were maintained in Eagle’s minimal essential medium (EMEM) with 5% fetal bovine serum (FBS, Gibco, Grand Island, NE, USA), L-glutamine (Gibco, Grand Island, USA), and 1% antibiotic (Gibco, Grand Island, USA). Human embryonic kidney (HEK) 293T cells and A549 cells were maintained in Dulbecco’s Modified Eagle Medium (DMEM) supplemented with 10% FBS (Gibco, L-glutamine (Gibco), and 1% antibiotic (Gibco).

### 2.2. Plasmid Construction and Virus Rescue

The wild-type (WT) CA09-M1, CA09-M2 genes, and CA09-M2-E79K genes were amplified from A/California/04/2009 H1N1 (CA09) (GenBank: FJ969513.1) viruses and subcloned to pCDNA3.0 expression vector with the FLAG tag fused to the N-terminus of the proteins, resulting in wild-type M2 (3FLAG-M2-WT), E79K M2 (3FLAG-M2-E79K), and wild-type M1 (3FLAG-M1-WT) for protein expression and viral budding assay. The A/swine/Texas/4199-2/1998(H3N2) (TX98) (GenBank: CY095678.1) M2 gene was cloned to pCDNA3.0 expression vector with the FLAG tag fused to the N-terminus of the proteins to obtain 3FLAG-H3N2M2, and then a HA tag was further fused to the C-terminus of the inserted H3N2-M2, H1N1-M2-WT, and H1N1M2-E79K genes, resulting in 3FLAG-H3N2M2-HA, 3FLAG-M2-E79K-HA, and 3FLAG-M2-WT-HA. To investigate which residue is responsible for the mobility change, CA09 M2 is mutated to H3N2 M2 with a site-mutagenesis kit (Yeasen, Shanghai, China). CA09-M2-5AA is M2 that contains the mutations of residues 11, 13, 14, 18, and 20; CA09-M2-3AA contains the mutations of residues 27, 28, and 31; CA09-M2 QE/RK contains residues 77 and 79; and CA09 E/K and CA09 Q/R contain single mutations. The wild-types CA09 (CA09-M2-WT) and CA09-M2-E79K were then rescued as previously described [24]. The reverse genetic system of CA09 and the plasmid with the full-length M gene of TX98 were generously provided by Dr. Qinfang Liu from the Shanghai Veterinary Research Institute [25,26]. The two viruses were propagated and titrated on MDCK cells. The sequences of the rescued viruses were confirmed with the wild-type virus by sequencing.

### 2.3. Protein Expression and Western Blotting

To evaluate the protein expression levels of M2 expression plasmids and budding efficiency, plasmids were transfected into cultured 293T cells and the supernatant was collected after transfection. Twenty-four hours post transfection, the cells were collected and lysed with RIPA cell lysis buffer (Yeasen). The cell lysates were sent to Western blotting assay by loading onto 12.5% sodium dodecyl sulfate (SDS)-polyacrylamide gel, and were then transferred to a PVDF membrane, followed by blocking in PBS buffer containing 5% skimmed milk. The proteins were detected by incubating with mouse anti-FLAG antibody or anti-HA antibody (Sigma-Aldrich, St. Louis, MO, USA) and then with horseradish peroxidase-conjugated anti-mouse antibody (Yeasen); they were then visualized with the ECL kit (Yeasen).

### 2.4. Growth Kinetic Study

To evaluate the viral growth kinetics in vitro, CA09-M2-WT and CA09-M2-E79K were inoculated on monolayer MDCK cultured in 12-well plates with a multiplicity of infection (MOI) of 0.001. The supernatants were collected at 12, 24, 36, and 48 h post inoculation (hpi), with each time point performed in triplicate. The samples were titrated on MDCK cells cultured in a 96-well plate to calculate TCID_50_/mL following the Reed and Muench method.

### 2.5. Animal Study

To evaluate the pathogenicity of the wild-type and mutant viruses, thirty-six 4-week-old female BALB/c mice were randomly allocated into three groups, each group containing 12 mice. Two groups were inoculated with CA09-M2-WT and CA09-M2-E79K, respectively, and one group with PBS as a control. The mice were intranasally infected with 50 µL of the 10^5.5^ TCID_50_ virus under slight anesthesia with CO_2_ [26,27]. This dose of virus can cause high mortality to mice, which makes it easy to distinguish the difference in pathogenicity of different viruses; the survival rates of the challenge doses of 10^4.5^ and 10^5.5^ TCID_50_ are shown in Figure S3. The mice were monitored daily for clinical signs, body weight, and survival rate until 14 days post infection (dpi). The mice who lost more than 25% of their original body weight were euthanized. At 3 and 5 dpi, three mice of each group were euthanized and necropsied for pathogenesis study. The mouse lungs were then collected, half of which were fixed in 4% paraformaldehyde for histopathology study; the other half were stored at −80 °C for viral titration and cytokine analysis.

### 2.6. Real Time RT-PCR to Evaluate Cytokines

Quantitative real-time polymerase chain reaction was employed to relatively quantify the cytokine levels of TNF-α, IL-6, IL-β, IL-10, IFN-β, IFN-γ, and IL-12 in the mouse lungs. An amount of 0.03 g of each mouse lung was homogenated in RNA isolator (Vazyme, Nanjing, China) and total RNA was extracted and transcribed with SuperScript^®^ III Reverse Transcriptase (Invitrogen, Carlsbad, CA, USA) to cDNA with transcriptase following the manufacture’s instructions (Vazyme). Briefly, the quantitative PCR was conducted in a 20 μL reaction volume containing 10 μL 2×ChamQ SYBR qPCR master mix (Vazyme), 0.4 μL of each primer (10 μM), 2 μL cDNA, and 7.2 μL RNase-Free water. The real-time PCR condition was set as follows: 95 °C for 30 s, then 40 cycles of 95 °C for 10 s, and then 60 °C for 30 s. The primers used in the study are listed in Table 1.

To determine if the M2 E79K mutation will affect the NLRP3 pathways in vitro, CA09-M2-WT and CA09-M2-E79K viruses were infected onto cultured 6-well A549 cells at MOI 0.01. Twenty-four hours post inoculation, the cells were lysed in RNA isolator. Total RNA was extracted and reverse transcribed into cDNA, as described above. The primers targeting the NLRP3 pathway was used as previously reported [28], and primers are listed in Table 1. The quantitative real-time RT PCR was performed as described above.

### 2.7. Viral Budding Assay

To explore if the E79K mutation will affect virus budding, 3FLAG-M2-WT (2 μg) or 3FLAG-M2-E79K (2 μg) plasmids were either transfected or co-transfected with 3FLAG-M1-WT into 293T cells cultured in a 100 mm dish, respectively. Forty-eight hours post transfection, the supernatants were harvested and centrifugated at 2000× *g* for 20 min to remove cell debris. The clarified supernatants were then centrifuged at 20,000× *g* for 2 h at 4 °C with a 20% (*w*/*v*) sucrose cushion (PBS) in a Beckman ultracentrifuge (Beckman Coulter, Fullerton, CA). The pellet was re-suspended in 60 μL of PBS on ice. The transfected cells were collected and lysed in 500 μL of lysis buffer (RIPA). Both budded particles and cell lysates were subjected to Western blotting assay. Both M1 and M2 proteins were detected with monoclonal mouse anti-FLAG antibody (Sigma-Aldrich).

### 2.8. Electric Microscopy for VLP and Virus Particle Observation

3FLAG-M2-WT or 3FLAG-M2-E79K were transfected on 293T cells to generate VLPs. Twenty-four hours post transfection, supernatants were collected and centrifuged at 2000× *g* for 20 min to remove the cell debris. MDCK cells were infected with CA09-M2-WT or CA09-M2-E79K viruses; 48 h post inoculation the supernatant was collected and centrifuged at 2000× *g* for 20 min to remove the cell debris. The clarified supernatants were ultra-centrifuged at 20,000× *g* and cushioned with 20% (*w*/*v*) sucrose. After centrifugation, the pellets were collected on ice; the virus particles were fixed in 4% paraformaldehyde at 37 °C overnight and then sent for electric microscopy. The service was provided by the Instrumental Analysis Center at Shanghai Jiao Tong University with Tecnai™ G2 spirit Biotwin (Thermo Fisher, Grand Island, NE, USA).

### 2.9. Mass Spectrometry

To explore if there is any post-translational modification (PTM) on M2 proteins, 3FLAG-M2-WT and 3FLAG-E79K-M2 plasmids were transfected on confluent 293T cells for 24 h, with 4 μg in each well. Cell lysate was collected with RIPA and incubated with 3FLAG-conjugated agarose overnight. The agarose was washed five times before the proteins were eluted with 0.1 M Gly-HCl at pH 3.5. The eluted proteins were sent to mass spectrometry to detect certain PTMs, including phosphorylation, methylation, acetylation, ubiquitination, SUMOylation, and fatty acylation. The service was provided by Hoogen biotech (Shanghai, China), and this study has been confirmed with two independent assays.

### 2.10. Circular Dichroism Spectra Analyze

To further explore the mechanism of the mobility difference between the two M2 proteins, the eukaryotic-expressed proteins were purified and then subjected to circular dichroism spectra analysis. The circular dichroism spectra were used to scan the secondary structure of the proteins. The dots data were connected into a line chart. The service was provided by the Instrumental Analysis Center at Shanghai Jiao Tong University with a circular dichroism spectrometer J-815 (JASCO, Tokyo, Japan).

### 2.11. Ethics Statement and Statistical Analysis

The animal experiment in this study was conducted in accordance with the guidelines of the Animal Care and Use Committee of Shanghai Jiao Tong University, and the animal study protocols were approved by Ethics Committee of Shanghai Jiao Tong University. The approval code is A-2020-056 and the approval date is 28 September 2020.

The virus titers and the real-time PCR results were analyzed by using analysis of variance (ANOVA) in GraphPad Prism version 5.0 (GraphPad software Inc., La Jolla, CA, USA); a *p*-value of 0.05 or less was considered significant.

## 3. Results

### 3.1. The M2 E79K Mutation Changed the Mobility Rate and Affected Viral Replication In Vitro

**Figure 1:**
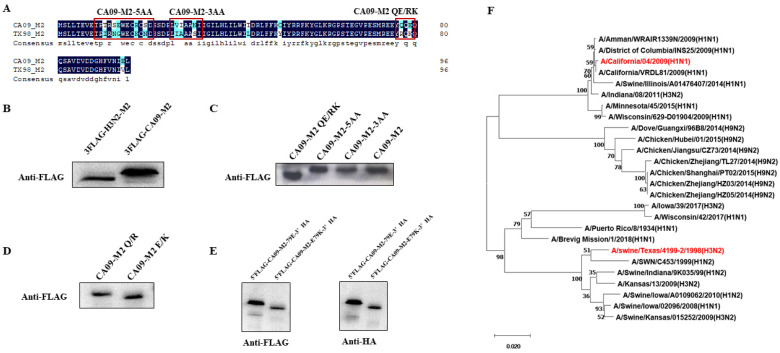
CA09 M2 E79K is responsible for the mobility rate change in SDS-PAGE assay. (**A**) The amino acid alignment between CA09 M2 and TX98 M2. The alignment is performed with DNAMAN software v5.2.2. (**B**) The 3FLAG-H3N2-M2 and 3FLAG-CA09-M2 protein detected by anti-FLAG antibody. The two proteins with the same amino acid number showed different mobility rates in SDS-PAGE. (**C**) Site-directed mutagenesis of different amino acids in the alignment with grouping: CA09-M2-5AA is CA09 M2 containing the mutation of H3N2 M2 at residues 11, 13, 14, 18, and 20; CA09-M2-3AA contains the mutation of residues 27, 28, and 31 from H3N2 M2; CA09-M2 QE/RK contains the mutations at residues 77 and 79. CA09-M2 QE/RK showed a smaller molecular weight compared with CA09 M2-WT. (**D**) Comparison of CA09 M2-Q77E and CA09 M2-E79K in Western blotting assay. (**E**) M2 proteins detected with FLAG tag fused to N-terminus end and HA tag fused to C-terminus. (**F**) Phylogenetic analysis of A/California/04/2009(H1N1) (marked in red, CA09) and A/swine/Texas/4199-2/1998(H3N2) (marked in red, TX98) M2 genes used in this study with neighbor-joining bootstrap method 1000 replications using MEGA-X software v10.2.6 [29].

The present study showed that the mobility rate of the Eurasian avian-like M2 protein of the 2009 pandemic H1N1 (CA09-M2-WT) is lower than that of the North America triple-reassortant H3N2-M2 in SDS-PAGE (Figure 1B). Based on the alignment, there were thirteen amino acid differences between the two M2 proteins, as shown in Figure 1A. To investigate the residue that determines the mobility rates, site-directed mutagenesis was performed on the CA09-M2-WT gene. The mutated genes were expressed and the mobility rates were examined. The Western blotting analysis showed that the CA09-M2 E79K mutation is responsible for the molecular weight change of the protein (Figure 1C,D). To investigate if this change was due to proteolysis, two tags were fused to the N-terminus and C-terminus of the CA09-M2-E79 and CA09-M2 E79K proteins. The results showed that both proteins with two tags were detected with the same molecular weight, indicating that the protein had not been proteolyzed (Figure 1E). The phylogenetic analysis of the CA09 and TX98 M2 genes are shown in Figure 1F.

**Figure 2:**
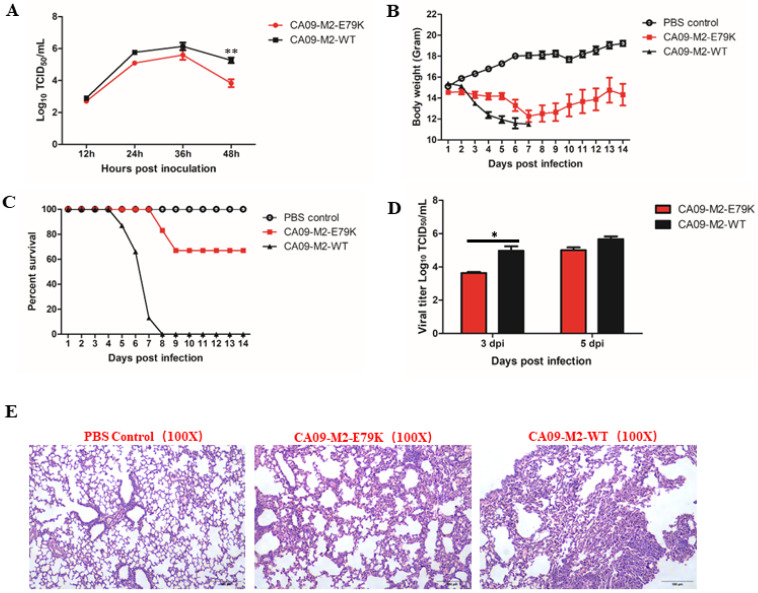
Study of CA09 virus with M2 E79K mutation in vitro and in vivo. (**A**) The virus growth kinetics on MDCK cells with MOI = 0.001. The study was performed independently in triplicate. (**B**) Mouse body weight data collected for 14 days after infected with CA09-M2-WT, CA09-M2-E79K, or PBS. (**C**) The survival rate of mice in CA09-M2-WT-, CA09-M2-E79K-, and PBS-inoculated groups. (**D**) The viral titers in mouse lungs collected on 3 dpi and 5 dpi. *: *p* < 0.05 and **: *p* < 0.01 are considered significant. (**E**) Histopathological examination of mouse lungs collected on 3 dpi. The tissue is stained with H&E and visualized with a magnification of 10 × 10.

The multi-alignment of M2 genes from GenBank showed that M2 E79 is highly conservative among Eurasia-origin swine influenza viruses, but not North-America swine influenza viruses. The frequencies of E79 in human and swine influenza viruses in M2 in North America before and after (H1N1)pdm09 has also been analyzed and is shown in Table 2. The results show that there are more viruses containing 79E in M2 of swine H1N2, swine H3N2, human H1N1, and human H3N2 subtypes after (H1N1)pdm09. There is no K in residue 79 in human H1N2 before or after 2009. The results indicate that M2 E79 is more viable in swine herds. To assess whether this mutation affected virus replication, the CA09-M2-E79K virus containing an E79K mutation on the M2 protein of CA09 and the wild-type CA09 (CA09-M2-WT) were rescued using a reverse genetic system. The two viruses were then inoculated on MDCK cells and their growth kinetics were evaluated. In Figure 2A, the results show that the CA09-M2-E79K replicated to a lower titer than the CA09-WT at 12, 24, and 36 hpi. The significant difference was observed only at 48 hpi, which suggests that the M2-E79K mutation has a limited effect on virus replication on cultured cells.

### 3.2. The M2-79E Is Essential for the Virulence of the CA09-WT Virus in Mouse Models

To evaluate the effect of M2-E79K on the viral pathogenicity in vivo, the CA09-M2-E79K and the CA09-M2-WT viruses were inoculated into mice intranasally. Both viruses caused clinical signs, such as ruffled fur, depression, loss of appetite, and loss of body weight. However, the CA09-M2-WT-infected mice showed more severe clinical symptoms than the CA09-M2-E79K-infected mice. The CA09-M2-E79K infection resulted in less body weight loss than the CA09-M2-WT virus to mice (Figure 2B). The CA09-M2-E79K caused only 30% mortality during the study, whilst the CA09-M2-WT virus resulted in 100% mortality by 7 dpi (Figure 2C). The viral titers in mouse lungs were analyzed to evaluate the replication ability of the viruses in vivo. The CA09-M2-WT replicated to higher titers than the mutant CA09-M2-E79K on both 3 and 5 dpi, with significant difference observed on 3 dpi (Figure 2D). The histopathological examinations showed that the CA09-M2-WT caused severe lesions (Figure 2E), such as infiltration with neutrophils of the alveolar lumen and lung interstitial substance, alveolar collapse, damage of the alveolar epithelium, and bronchiolitis inflammatory exudation and collapse. On the other hand, the CA09-M2-E79K virus that infected mouse lungs showed less severe lesions compared with the CA09-M2-WT virus, such as neutrophil infiltration of lung interstitial substance, damage of alveolar, and bronchiolitis partial collapse.

### 3.3. The CA09-M2-E79K Induced Less Inflammatory Response and NLRP3 Inflammasome Activation

**Figure 3:**
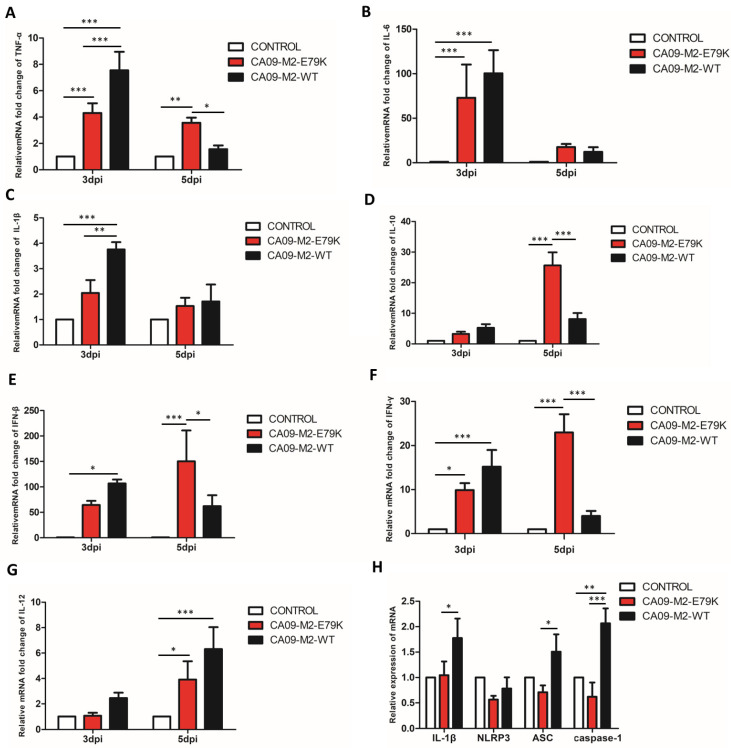
The mRNA levels of cytokines in mouse lungs collected on 3 and 5 dpi. The data were obtained with real-time RT PCR and calculated with 2^−∆∆Ct^. (**A**) TNF-α, (**B**) IL-6, (**C**) IL-1β, (**D**) IL-10, (**E**) IFN-β, (**F**) IFN-γ, (**G**) IL-12, (**H**) The mRNA levels of the compartment of the NLRP3 complex IL-1β, NLRP3, ASC, and caspase-1 were evaluated with real-time RT PCR on cells infected with CA09-M2-WT and CA09-M2-E79K viruses, respectively. *: *p* < 0.05, **: *p* < 0.01, and ***: *p* < 0.001 are considered significant.

The levels of various inflammatory cytokines, including TNF-α, IL-6, IL-β, IL-10, IFN-β, IFN-γ, and IL-12, were tested in the lungs of the infected mice. In Figure 3, itcan be seen that, on 3 dpi, mice inoculated with CA09-M2-E79K exhibited lower levels of pro-inflammatory cytokines. All the tested cytokines in the CA09-M2-E79K group showed lower levels compared with those in the CA09-M2-WT group, and significances were observed on TNF-α, IL-1β, and IFN-β. Since IL-1β is secreted after activation of NLRP3 inflammasome, the results indicated that the CA09-M2-E79K activated NLRP3 inflammasome less efficiently than CA09-M2-WT. Interestingly, both viruses caused similar levels of IFN-β and IFN-γ on 3 dpi, but on 5 dpi CA09-M2-E79K induced higher levels of both IFN-β and IFN-γ than CA09-M2-WT, and significantly higher levels of the anti-inflammatory cytokine IL-10 compared with CA09-M2-WT. The results indicate that CA09-M2-E79K induced less inflammation than CA09-M2-WT on 3 dpi and antagonized the IFN response less efficiently than CA09-M2-WT on 5 dpi, which may lead to lower virulence and faster clearance of the virus in mice compared with CA09-M2-WT.

Since the M2 protein has been reported to be related to NLRP3 inflammasome activity [22], we investigated if the M2 E79K mutation affects the virulence of CA09-M2-E79K through the NLRP3 pathway. The NLRP3 inflammasome component transcription levels were evaluated in the cell that was infected by the CA09-M2-WT or CA09-M2-E79K virus. The results showed that the mRNA levels of ASC, caspase-1, NLRP3, and IL-1β in the CA09-M2-E79K-infected cells were lower than those of the CA09-M2-WT-infected cells (Figure 3H). These results suggest that the M2 E79K mutation affects NLRP3 inflammasome complex activation.

### 3.4. The M2-E79K Affected VLP Budding Ability

**Figure 4:**
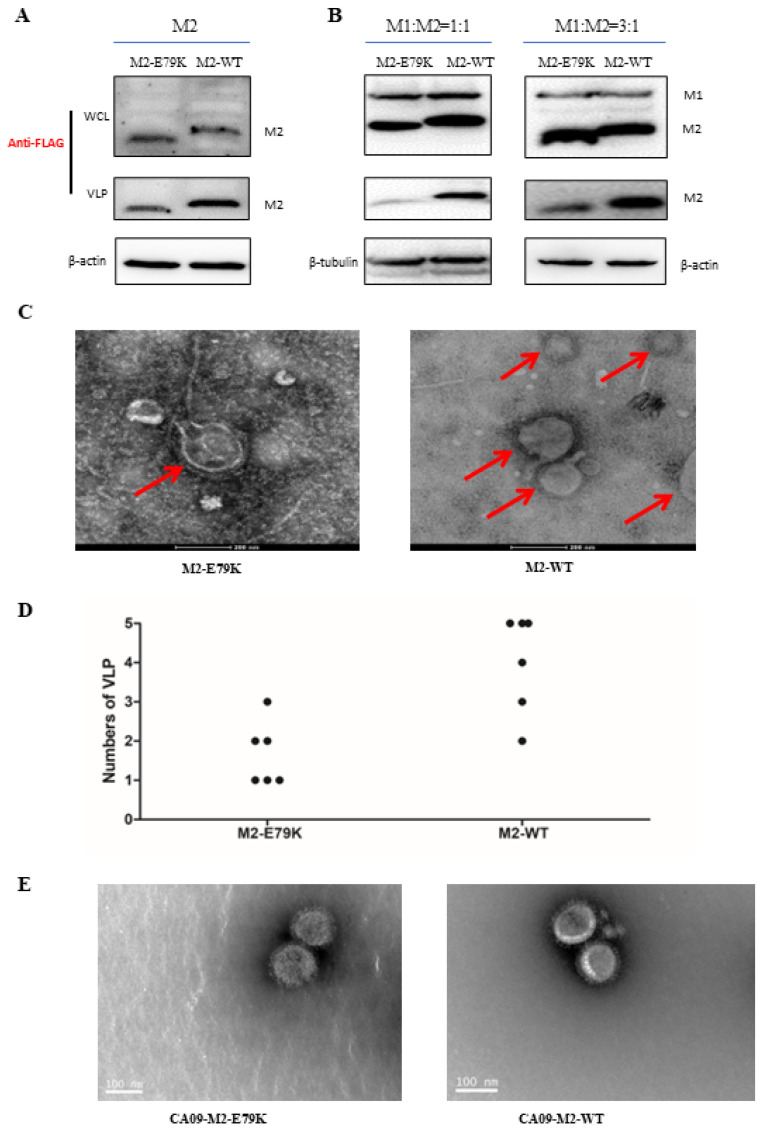
(**A**) M2 levels in WCL or VLP in M2 transfection group. (**B**) M2 and M1 levels in WCL and M2 levels in VLP in M1:M2 = 1:1 and 3:1 co-transfection groups. (**C**) VLPs obtained from M2-E79K- or M2-WT-transfected supernatants. The particles were observed under electronic microscope. The scale bar is 200 nm. The red arrows indicate VLPs formed by M2 proteins. (**D**) The analysis of possible VLP numbers in each view, n = 6. (**E**) The virions in CA09-M2-E79K- and CA09-M2-WT-infected cell supernatant observed with EM. The scale bar is 100 nm.

M2 protein is a membrane protein that plays a crucial role in viral budding. Previous studies have shown that the M2 protein is capable of budding on its own. To investigate the impact of the M2 E79K mutation on budding efficiency, the budding ability of M2 protein and M2 in combination with M1 were examined. In the budding assay, it was found that both M2-WT and M2-E79K were efficiently expressed at similar levels in the whole cell lysate (WCL). Instead, in the VLP samples, the M2-E79K budded less efficiently than M2-WT (Figure 4A). To further explore whether M2 budding efficiency is dependent on M1, 3FLAG-M2-WT or 3FLAG-M2-E79K were co-transfected with 3FLAG-M1. In the M1:M2 = 1:1 group, as shown in Figure 4B, the 3FLAG-M2-E79K and 3FLAG-M1-WT were expressed at similar levels with 3FLAG-M1 co-transfection. Interestingly, the 3FLAG-M2-WT was successfully budded to the medium, but a much smaller amount of the 3FLAG-M2-E79K was budded. Similar results were observed in the M1:M2 = 3:1 group. The results indicated that, with or without the help of the M1 protein, the M2-E79K buds less efficiently than M1-WT.

In order to better understand the impact of the M2-E79K mutation on viral budding efficiency and morphology, electron microscopy was employed to observe the budded VLPs. Cells were transfected with either 3FLAG-M2-E79K or 3FLAG-M2-WT, and the supernatants were collected and ultracentrifuged to concentrate the VLPs. The results, as shown in Figure 4C, illustrated that there were more VLPs with intact morphology in the 3FLAG-M1-WT group than in the 3FLAG-M2-E79K group. In addition, the numbers of VLP in each view were counted and are shown in Figure 4D, with *n* = 6. This indicates that the budding ability of the virus is affected by the M2 E79K mutation, resulting in less VLPs and less intact viral morphology. To observe the virion budding, an EM study was employed to better elucidate the effect of M2 proteins on viral budding and morphology. The viruses budded to the supernatant with similar spherical shapes and similar sizes of approximately 100 nm (Figure 4E). Thus, the M2 E79K mutation does not affect virion morphology.

### 3.5. The M2-E79K Might Affect the Structure of M2 Protein

**Figure 5:**
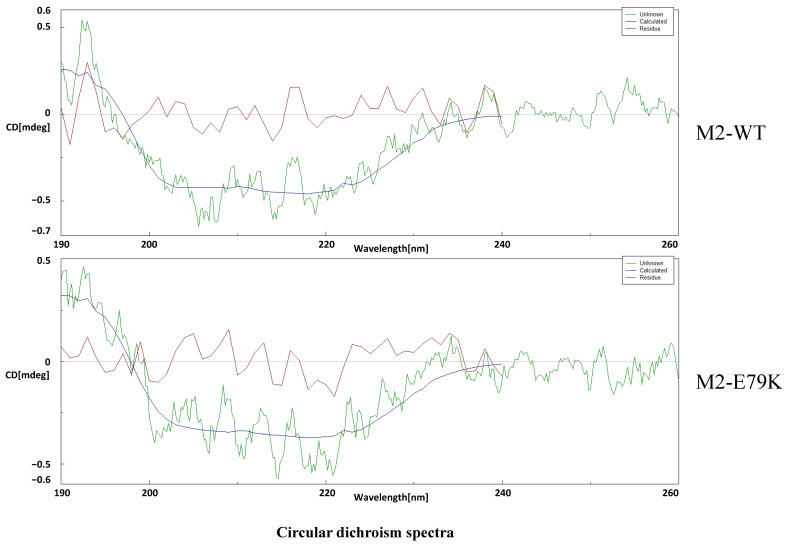
Protein structure analysis of CA09 M2-WT and M2-E79K proteins. Circular dichroism spectra analysis of M2-WT and M2-E79K proteins expressed on 293T cells.

The PTM of M2-WT and M2-E79K was screened using mass spectrometry and software prediction. Mass spectrometry analysis did not identify any possible modifications on this residue, including phosphorylation, acetylation, methylation, ubiquitination, SUMOylation, and fatty acylation; the results are shown in Table 3. In addition, the structures of M2-WT and M2-E79K were predicted using software SWISS-MODEL (Swiss Institute of Bioinformatics, Biozentrum, Switzerland) and Alpha fold 2 (DeepMind, London, UK), and no notable differences were found (data are shown in Supplementary Material, Figures S1 and S2). To further evaluate the structures, circular dichroism spectra was employed. The results showed that the secondary structures of the M2-WT and M2-E79K were slightly different based on the difference of the absorption coefficient at wavelengths between 190 and 200 μm (Figure 5), which suggests that the secondary structure of M2-E79K is different from M2-WT. These findings suggest that the mutation may have altered the secondary structure of the protein, which results in a different mobility rate in SDS-PAGE.

## 4. Discussion

The 2009, the H1N1 pandemic led to the global spread of the virus to different countries and animal species, resulting in variant viruses with diverse gene combinations. Notably, almost all of these variant viruses contained M genes from the (H1N1)pdm09 strain [12,30]. As a result, a lineage of H1N1, H1N2, and H3N2 viruses containing the (H1N1)pdm09 M gene emerged and became established in both swine herds and human populations. Studies have shown that the variant H3N2 swine influenza viruses, which contain the M gene from (H1N1)pdm09, can infect humans and cause deaths with limited human-to-human transmission [13,31]. The reason for the prevalence of the M gene from (H1N1)pdm09 in the variant viruses remains unclear. Several studies have focused on whether M1 or M2 plays a more significant role in the pathogenicity of the viruses. Jianmei [32] reported that both M1-41V and M2-27 contribute to replication and pathogenicity. Our previous study indicated that both M1 and M2 of the CA09 strain affects the virulence when a gene segment is exchanged with the H3N2 swine influenza virus [26]. In the present study, we focused on the M2 protein of the (H1N1)pdm09 virus and its role in pathogenicity. Our findings suggest that the E79K mutation in M2 greatly attenuated the virus, which highlights the importance of M2 function in influenza virus pathogenicity.

In the mouse study, the CA09-M2-E79K virus induced less pro-inflammatory cytokines. On the one hand, although M2-E79K affects the budding ability of the virus, it has a limited effect on viral replication in vitro. However, it may affect the progeny virus release in vivo. On the other hand, the cytokine induction is related to local inflammation, which is initiated by inflammasome. Influenza virus M2 was reported as being associated with NLRP3 inflammasome activation [33]. In the present study, the CA09-M2-E79K reduced the levels of caspase-1, ASC, NLRP3, and IL-1β, indicating a less efficient inflammasome activation. Therefore, the possible reason that the CA09-M2-E79K virus induced less pro-inflammatory cytokine is that it induces inflammasome maturation less efficiently. Meanwhile, the CA09-M2-E79K virus has been found to induce more IFN-β and IFN-γ than CA09-M2-WT on 5 dpi; however, the mechanism is unclear regarding whether the CA09-M2-E79K induces more IFNs or if the CA09-M2-WT antagonizes the IFN response more efficiently. The influenza NS1 gene has been for a long time considered the IFN antagonist, and there are only a few studies that have reported that the influenza virus M protein has the function of IFN antagonism [34]. Notably, the present study infers that the M2 protein may be related to IFN response, which is in agreement with that study.

The M2 protein contains the 97 amino acids protein that is composed of extracellular domain (24 amino acids), transmembrane domain (19 amino acids), and 54 cytoplasmic domain (54 amino acids) [19]. The cytoplasmic domain is highly conserved and is the longest cytoplasmic domain of the transmembrane proteins of influenza A viruses, which is reported as being important for assembly and budding but is still not well understood [35]. Its deletion has been shown to negatively affect viral replication, as evidenced by the failure to propagate the viruses lacking this domain [36]. Additionally, amino acid substitutions found in the M2 cytoplasmic tail or in the M1 protein of mutants selected by a monoclonal antibody against the M2 ectodomain suggest a possible interaction between the cytoplasmic domain of M2 and the M1 protein [37]. The M2 cytoplasmic domain, where the 79th amino acid is localized, plays a role in coordinating the efficient packaging of genome segments into influenza virus particles. A study indicated that the M2 74-79 residues are critical to virus budding [38]. The present data are in agreement with the study showing that M2-WT buds more efficiently than M2-E79K, regardless of the presence of M1. These findings provide new insights into the role of M2 proteins in viral budding and may have implications for the development of antiviral drugs. In this study, the FLAG tag was fused to the M2 protein, which may affect the function of M2. In the budding assay, the M2 protein can form VLP, which indicates that the FLAG tag did not affect the budding function of M2. However, an anti-M2 primary antibody should be used to further confirm the results. Due to this limitation, the virion morphology was then observed. The virion sizes and morphology are similar between the two viruses, which indicates that the M2 protein will cooperate with other viral proteins in assembly and budding. Therefore, the M2 E79K does not have much effect on morphology.

Based on previous studies, most of the structure-elucidating studies about influenza M2 proteins are focused on the ion channel domain, and the sequences they used contain E79 not K79 [39,40]. In the present study, the post-translational modifications were screened and underwent protein hydrolyzation, but none of them happens. Most of the time, the modifications happen on K and not on E. Thus, we speculated that the electric charge brought by amino acid change may change the secondary structure. The amino acid K is positively charged and E is negatively charged. Thus, we speculate that the electric charge brought by amino acid change may change the secondary structure. The in-silico predictions have been carried out to elucidate the possible structure change. However, the two predictions gave different results. In both methods, the M2 E79K mutation did not cause an obvious structure change, whereas, in the experiment, this mutation causes a large mobility change. Thus, the circular dichroism spectra were employed to study the structures of M2 experimentally, which showed a difference in the secondary structures.

Taken together, our results suggest that the M2 cytoplasmic tail plays a crucial role in virus pathogenicity, specifically in terms of proinflammatory response through the NLRP3 pathway. The E79K mutation significantly affects virus budding ability in vitro, and CA09-M2-E79K replicates less efficiently than the wild-type virus in vivo. The different mobility rates caused by the E79K mutation may result from the changes of the secondary structures of the proteins.

## Figures and Tables

**Table 1:** Primer pairs used in quantitative real-time RT-PCR for specific target genes.

Gene	Forward Primers	Reverse Primers
Human IL-1β	5′-CTGATGGCCCTAAACAGATGAAG-3′	5′-GGTCGGAGATTCGTAGCAGCTGGAT-3′
Human NLRP3	5′-CTTCTCTGATGAGGCCCAAG-3′	5′-GCAGCAAACTGGAAAGGAAG-3′
Human ASC	5′-ATCCAGGCCCCTCCTCAGT-3′	5′-GTTTGTGACCCTCGCGATAAG-3′
Human caspase-1	5′-ATCCGTTCCATGGGTGAAGGTACA-3′	5′-CAAATGCCTCCAGCTCTGTA-3′
Human GAPDH	5′-GTCAGTGGTGGACCTGACCT-3′	5′-AGGGGTCTACATGGCAACTG-3′
Mouse IFN-γ	5′-TGGTGGTGATGTCTACACTCCG-3′	5′-CGAGTTATTTGTCATTCGGGTGT-3′
Mouse IL-1β	5′-CACCTGGTACATCAGCACCTCAC-3′	5′-CATCAGAAACAGTCCAGCCCATAC-3′
Mouse TNF-α	5′-CGATGAGGTCAATCTGCCCA-3′	5′-CCAGGTCACTGTCCCAGCATC-3′
Mouse IL-6	5′-GAGGATACCACTCCCAACAGACC-3′	5′-AAGTGCATCGTTGTTCATACA-3′
Mouse IL-10	5′-GGTTGCCAAGCCTTATCGGA-3′	5′-ACCTGCTCCACTGCCTTGCT-3′
Mouse IL-12	5′-CCACCCTTGCCCTCCTAAAC-3′	5′-GTTTTTCTCTGGCCGTCTTCA-3′
Mouse GAPDH	5′-GAAGGGCATCTTGGGCTCACT-3′	5′-GGTGGGTGGTCCAGGGTTTCTTA-3′

**Table 2:** Sequence alignment of residue E79 in M2 of human and swine influenza viruses before and after (H1N1)pdm09 in North America.

Before (H1N1)pdm09			After (H1N1)pdm09 (Contains 2009)		
Subtype	Human	Swine	Subtype	Human	Swine
H1N1	98.7% ^c^ (891 ^a^/903 ^b^)	99.7% (302/303)	H1N1	99.9% (13,434/13,440)	93% (207/2978)
H1N2	100% (23/23)	1.6% (1/61)	H1N2	100% (7/7)	91.5% (2626/2869)
H3N2	99.8% (1287/1289)	10.9% (14/128)	H3N2	99.9% (6/25,503)	87.5% (2700/3085)

^a^: The number of M2 sequence contains E on residue 79. ^b^: The total number of M2 sequences in certain subtypes. ^c^: The percentage of M2 sequences contains E on residue 79 in certain subtype.

**Table 3:** The mass-spectrometry results of CA09 M2-WT protein. (In Sequence, & is not a methylation modification site, T is a phosphorylation modification site, and @ is a methylation modification).

FileScan	Sequence	MH+	Diff (MH+)
R1477, 18396	-.M&SLLT^E@VETPTR.S	1486.68588	−0.01987
Charge	Score	Reference	PI
3	2.45	r1477	4.53
Modification			
15.994919 Oxidation (M); 79.966324 Phospho (ST); 14.015656 Methyl (DE)			

## Data Availability

Data is available upon request.

## Supplementary Materials

The following supporting information can be downloaded at https://www.mdpi.com/article/10.3390/v15122365/s1, Figure S1: The protein structure predictions by SWISS-MODEL; Figure S2: The protein structure predictions by Alphafold2; Figure S3: The survival rates of mice inoculated with different doses of CA09 influenza virus.

## References

[1] Szewczyk B., Bienkowska-Szewczyk K., Krol E. (2014). Introduction to molecular biology of influenza a viruses. Acta Biochim. Pol..

[2] Klenk H.D. (2013). Evolution and infection biology of new influenza A viruses with pandemic potential. Bundesgesundheitsblatt Gesundheitsforschung Gesundheitsschutz.

[3] Bouvier N.M., Palese P. (2008). The biology of influenza viruses. Vaccine.

[4] Tong S., Li Y., Rivailler P., Conrardy C., Castillo D.A., Chen L.M., Recuenco S., Ellison J.A., Davis C.T., York I.A. (2012). A distinct lineage of influenza A virus from bats. Proc. Natl. Acad. Sci. USA.

[5] Tong S., Zhu X., Li Y., Shi M., Zhang J., Bourgeois M., Yang H., Chen X., Recuenco S., Gomez J. (2013). New world bats harbor diverse influenza A viruses. PLoS Pathog..

[6] Sutton T.C. (2018). The Pandemic Threat of Emerging H5 and H7 Avian Influenza Viruses. Viruses.

[7] Dawood F.S., Jain S., Finelli L., Shaw M.W., Lindstrom S., Garten R.J., Gubareva L.V., Xu X., Bridges C.B., Novel Swine-Origin Influenza A (H1N1) (2009). Emergence of a novel swine-origin influenza A (H1N1) virus in humans. N. Engl. J. Med..

[8] Fowlkes A.L., Arguin P., Biggerstaff M.S., Gindler J., Blau D., Jain S., Dhara R., McLaughlin J., Turnipseed E., Meyer J.J. (2011). Epidemiology of 2009 pandemic influenza A (H1N1) deaths in the United States, April–July 2009. Clin. Infect. Dis..

[9] Smith G.J., Vijaykrishna D., Bahl J., Lycett S.J., Worobey M., Pybus O.G., Ma S.K., Cheung C.L., Raghwani J., Bhatt S. (2009). Origins and evolutionary genomics of the 2009 swine-origin H1N1 influenza A epidemic. Nature.

[10] Lakdawala S.S., Lamirande E.W., Suguitan A.L., Wang W., Santos C.P., Vogel L., Matsuoka Y., Lindsley W.G., Jin H., Subbarao K. (2011). Eurasian-origin gene segments contribute to the transmissibility, aerosol release, and morphology of the 2009 pandemic H1N1 influenza virus. PLoS Pathog..

[11] Ma W., Liu Q., Bawa B., Qiao C., Qi W., Shen H., Chen Y., Ma J., Li X., Webby R.J. (2012). The neuraminidase and matrix genes of the 2009 pandemic influenza H1N1 virus cooperate functionally to facilitate efficient replication and transmissibility in pigs. J. Gen. Virol..

[12] Rajao D.S., Walia R.R., Campbell B., Gauger P.C., Janas-Martindale A., Killian M.L., Vincent A.L. (2017). Reassortment between Swine H3N2 and 2009 Pandemic H1N1 in the United States Resulted in Influenza A Viruses with Diverse Genetic Constellations with Variable Virulence in Pigs. J. Virol..

[13] Jhung M.A., Epperson S., Biggerstaff M., Allen D., Balish A., Barnes N., Beaudoin A., Berman L., Bidol S., Blanton L. (2013). Outbreak of variant influenza A(H3N2) virus in the United States. Clin. Infect. Dis..

[14] Campbell P.J., Danzy S., Kyriakis C.S., Deymier M.J., Lowen A.C., Steel J. (2014). The M segment of the 2009 pandemic influenza virus confers increased neuraminidase activity, filamentous morphology, and efficient contact transmissibility to A/Puerto Rico/8/1934-based reassortant viruses. J. Virol..

[15] Campbell P.J., Kyriakis C.S., Marshall N., Suppiah S., Seladi-Schulman J., Danzy S., Lowen A.C., Steel J. (2014). Residue 41 of the Eurasian avian-like swine influenza a virus matrix protein modulates virion filament length and efficiency of contact transmission. J. Virol..

[16] Zhu J., Jiang Z., Liu J. (2021). The matrix gene of pdm/09 H1N1 contributes to the pathogenicity and transmissibility of SIV in mammals. Vet. Microbiol..

[17] Wu C.Y., Jeng K.S., Lai M.M. (2011). The SUMOylation of matrix protein M1 modulates the assembly and morphogenesis of influenza A virus. J. Virol..

[18] Rossman J.S., Lamb R.A. (2011). Influenza virus assembly and budding. Virology.

[19] Pielak R.M., Chou J.J. (2011). Influenza M2 proton channels. Biochim. Biophys. Acta.

[20] Martyna A., Rossman J. (2014). Alterations of membrane curvature during influenza virus budding. Biochem. Soc. Trans..

[21] Coates B.M., Staricha K.L., Ravindran N., Koch C.M., Cheng Y., Davis J.M., Shumaker D.K., Ridge K.M. (2017). Inhibition of the NOD-Like Receptor Protein 3 Inflammasome Is Protective in Juvenile Influenza A Virus Infection. Front. Immunol..

[22] Ichinohe T., Pang I.K., Iwasaki A. (2010). Influenza virus activates inflammasomes via its intracellular M2 ion channel. Nat. Immunol..

[23] Choudhury S.K.M., Ma X., Abdullah S.W., Zheng H. (2021). Activation and Inhibition of the NLRP3 Inflammasome by RNA Viruses. J. Inflamm. Res..

[24] Qiao C., Liu Q., Bawa B., Shen H., Qi W., Chen Y., Mok C.K., Garcia-Sastre A., Richt J.A., Ma W. (2012). Pathogenicity and transmissibility of reassortant H9 influenza viruses with genes from pandemic H1N1 virus. J. Gen. Virol..

[25] Liu Q.F., Qiao C.L., Marjuki H., Bawa B., Ma J.Q., Guillossou S., Webby R.J., Richt J.A., Ma W.J. (2012). Combination of PB2 271A and SR Polymorphism at Positions 590/591 Is Critical for Viral Replication and Virulence of Swine Influenza Virus in Cultured Cells and. J. Virol..

[26] Xie L., Xu G., Xin L., Wang Z., Wu R., Wu M., Cheng Y., Wang H., Yan Y., Ma J. (2021). Eurasian Avian-like M1 Plays More Important Role than M2 in Pathogenicity of 2009 Pandemic H1N1 Influenza Virus in Mice. Viruses.

[27] Leea J.W., Yu H., Li Y.H., Ma J.J., Lang Y.E., Duff M., Henningson J., Liu Q.F., Li Y.H., Nagy A. (2017). Impacts of different expressions of PA-X protein on 2009 pandemic H1N1 virus replication, pathogenicity and host immune responses. Virology.

[28] Gu N.Y., Kim J.H., Han I.H., Im S.J., Seo M.Y., Chung Y.H., Ryu J.S. (2016). Trichomonas vaginalis induces IL-1beta production in a human prostate epithelial cell line by activating the NLRP3 inflammasome via reactive oxygen species and potassium ion efflux. Prostate.

[29] Kumar S., Stecher G., Li M., Knyaz C., Tamura K. (2018). MEGA X: Molecular Evolutionary Genetics Analysis across Computing Platforms. Mol. Biol. Evol..

[30] He P., Wang G., Mo Y., Yu Q., Xiao X., Yang W., Zhao W., Guo X., Chen Q., He J. (2018). Novel triple-reassortant influenza viruses in pigs, Guangxi, China. Emerg. Microbes Infect..

[31] Epperson S., Jhung M., Richards S., Quinlisk P., Ball L., Moll M., Boulton R., Haddy L., Biggerstaff M., Brammer L. (2013). Human infections with influenza A(H3N2) variant virus in the United States, 2011–2012. Clin. Infect. Dis..

[32] Yang J., Zhang P., Huang M., Qiao S., Liu Q., Chen H., Teng Q., Li X., Zhang Z., Yan D. (2021). Key Amino Acids of M1-41 and M2-27 Determine Growth and Pathogenicity of Chimeric H17 Bat Influenza Virus in Cells and in Mice. J. Virol..

[33] Chen I.Y., Ichinohe T. (2015). Response of host inflammasomes to viral infection. Trends Microbiol..

[34] Wang R., Zhu Y., Lin X., Ren C., Zhao J., Wang F., Gao X., Xiao R., Zhao L., Chen H. (2019). Influenza M2 protein regulates MAVS-mediated signaling pathway through interacting with MAVS and increasing ROS production. Autophagy.

[35] Tobler K., Kelly M.L., Pinto L.H., Lamb R.A. (1999). Effect of cytoplasmic tail truncations on the activity of the M(2) ion channel of influenza A virus. J. Virol..

[36] McCown M.F., Pekosz A. (2006). Distinct domains of the influenza a virus M2 protein cytoplasmic tail mediate binding to the M1 protein and facilitate infectious virus production. J. Virol..

[37] Zebedee S.L., Lamb R.A. (1989). Growth restriction of influenza A virus by M2 protein antibody is genetically linked to the M1 protein. Proc. Natl. Acad. Sci. USA.

[38] Iwatsuki-Horimoto K., Horimoto T., Noda T., Kiso M., Maeda J., Watanabe S., Muramoto Y., Fujii K., Kawaoka Y. (2006). The cytoplasmic tail of the influenza A virus M2 protein plays a role in viral assembly. J. Virol..

[39] Thomaston J.L., Alfonso-Prieto M., Woldeyes R.A., Fraser J.S., Klein M.L., Fiorin G., DeGrado W.F. (2015). High-resolution structures of the M2 channel from influenza A virus reveal dynamic pathways for proton stabilization and transduction. Proc. Natl. Acad. Sci. USA.

[40] Acharya R., Carnevale V., Fiorin G., Levine B.G., Polishchuk A.L., Balannik V., Samish I., Lamb R.A., Pinto L.H., DeGrado W.F. (2010). Structure and mechanism of proton transport through the transmembrane tetrameric M2 protein bundle of the influenza A virus. Proc. Natl. Acad. Sci. USA.

