# Supplementary material for: The Mobility of Eurasian Avian-like M2 Is Determined by Residue E79 Which Is Essential for Pathogenicity of 2009 Pandemic H1N1 Influenza Virus in Mice
Source: Viruses. 2023 Nov 30;15(12):2365. doi: 10.3390/v15122365 (PMC10747126; doi:10.3390/v15122365)
Supplement: Supplementary file 1 [file viruses-15-02365-s001.zip › viruses-2652832-supplementary.pdf]

Figure S1

SWISS-MODEL prediction

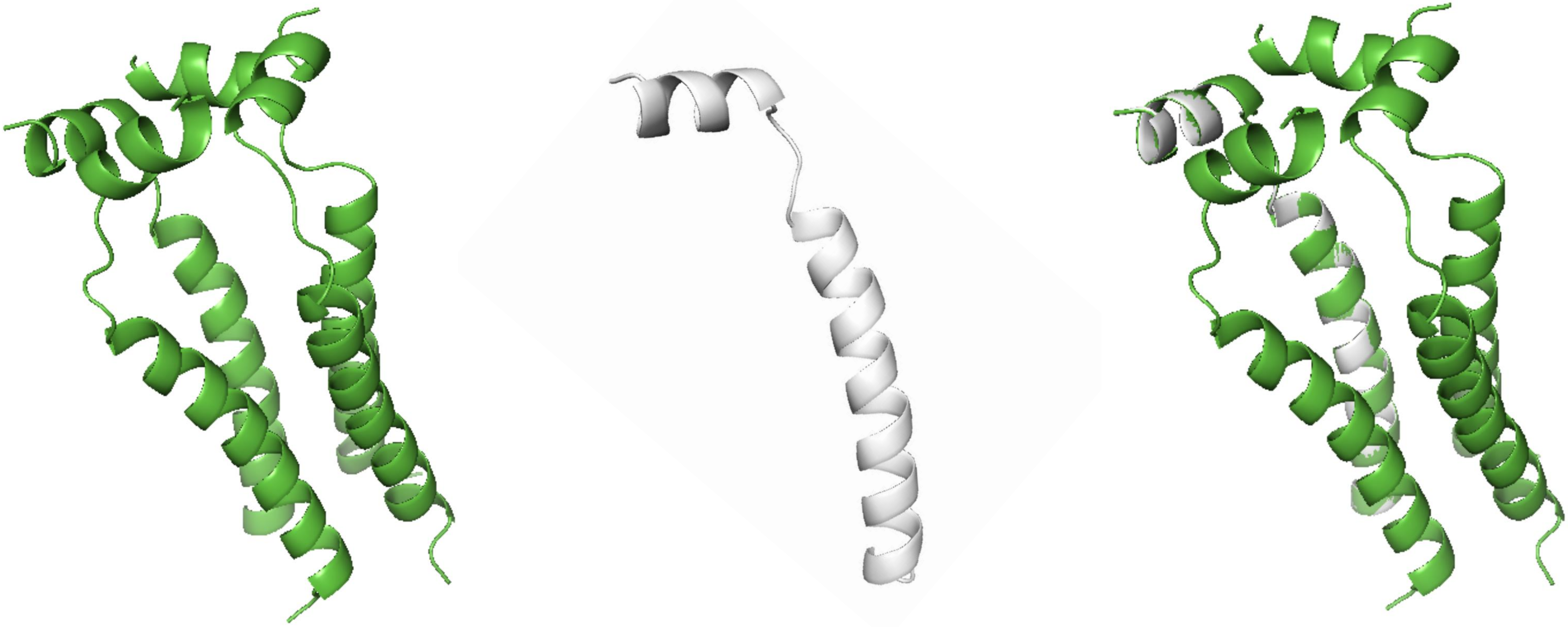

M2-E79K M2-WT Pymol overlap

Figure S2

Alphafold2 prediction

M2-E79K

M2-WT

Pymol overlap

M2-79: red spots

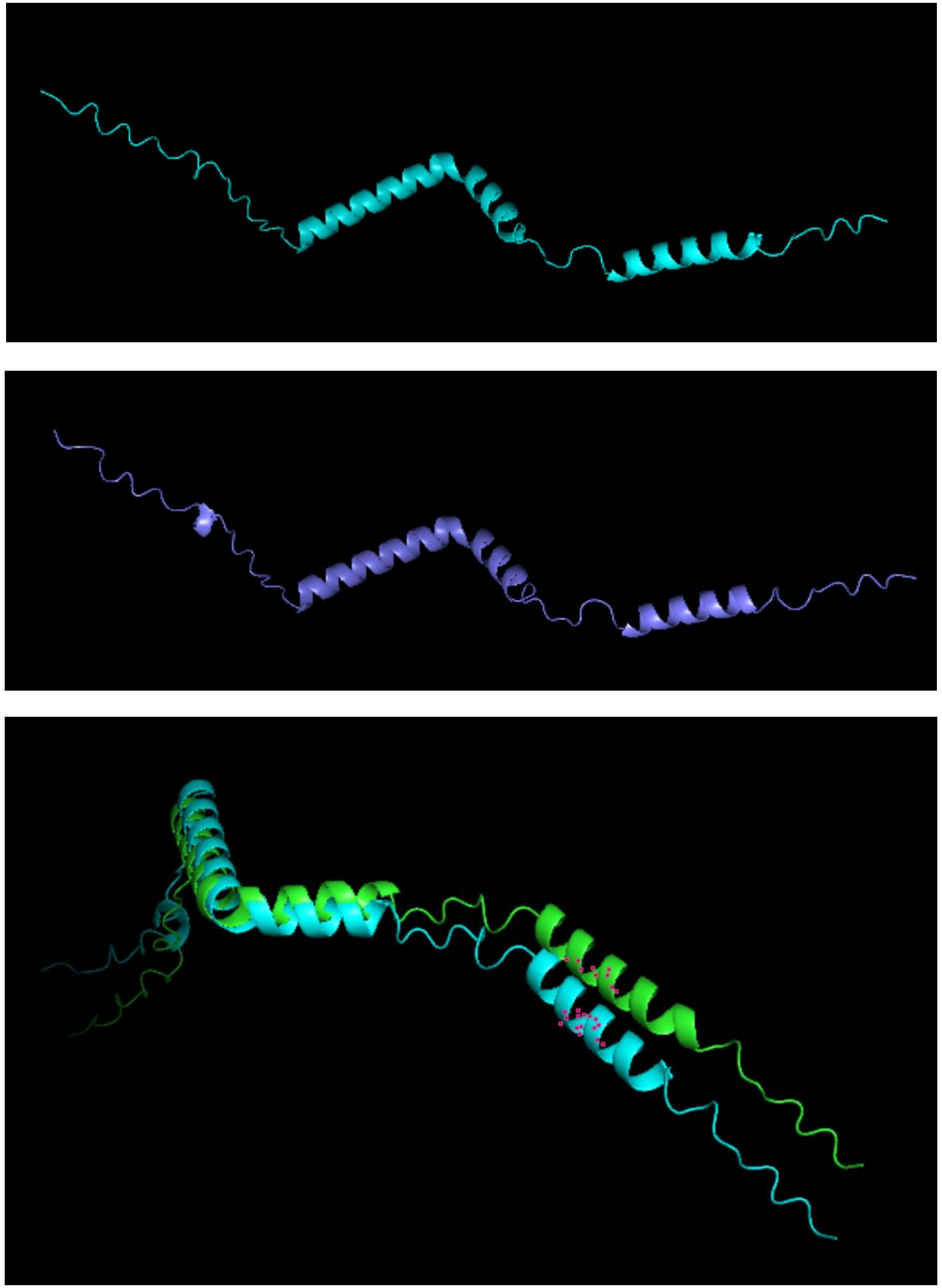

Figure S3

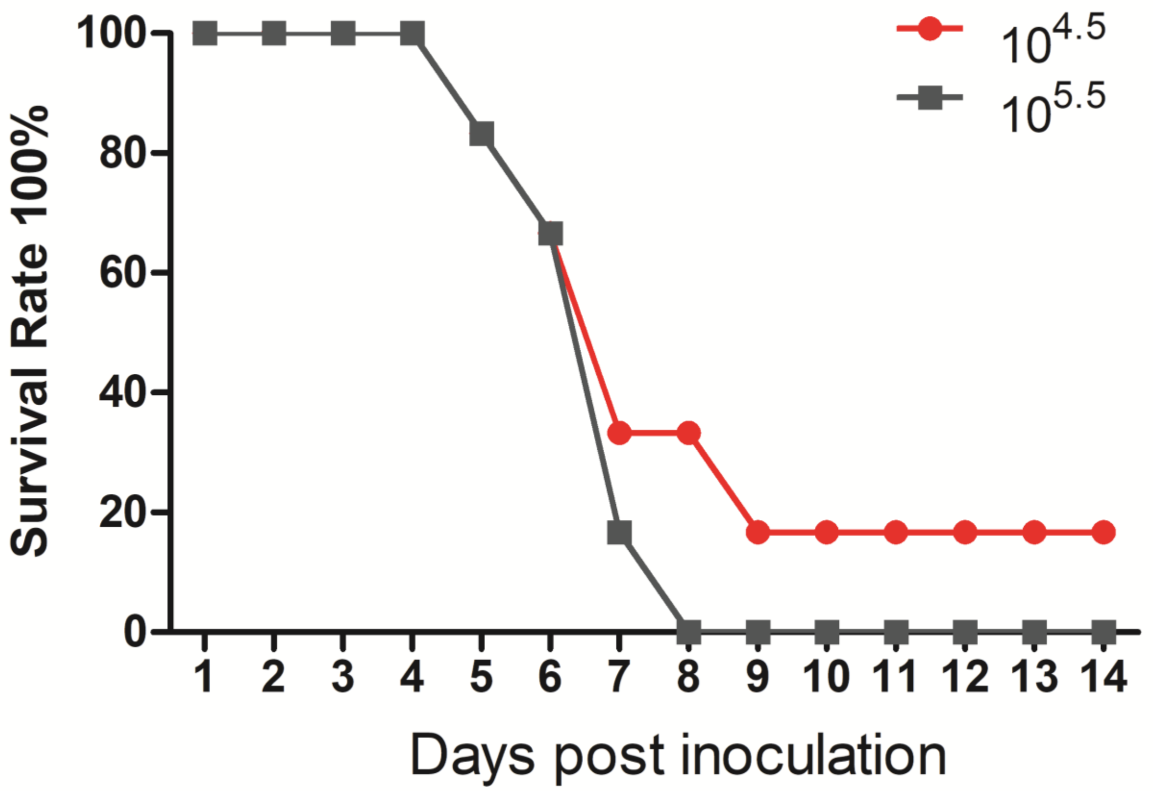

The survival rates of mice inoculated with different doses of CA09 influenza virus. Mice were intranasally challenged with $10^{4.5}$ $TCID_{50}$ and $10^{5.5}$ $TCID_{50}$. The dose of $10^{5.5}$ $TCID_{50}$ resulted in 100% mortality, whereas 16.7% mice survived with the challenge dose of $10^{4.5}$ $TCID_{50}$.
